# Chemotherapy for anaplastic thyroid cancer using docetaxel and cisplatin: report of eight cases

**DOI:** 10.1007/s00595-013-0751-x

**Published:** 2013-10-14

**Authors:** Akira Seto, Iwao Sugitani, Kazuhisa Toda, Kazuyoshi Kawabata, Shunji Takahashi, Takashi Saotome

**Affiliations:** 1 Division of Head and Neck, Cancer Institute Hospital, 3-8-31 Ariake, Koto-ku, Tokyo, 135-8550 Japan; 2 Department of Medical Oncology, Cancer Institute Hospital, 3-8-31 Ariake, Koto-ku, Tokyo, 135-8550 Japan; 3 Division of Endocrine Surgery, Department of Surgery, Nippon Medical School, 1-1-5 Sendagi, Bunkyo-ku, Tokyo, 113-8603 Japan; 4 Department of Medical Oncology, Matsudo City Hospital, 4005 Kamihongo, Matsudo, Chiba 271-8511 Japan

**Keywords:** Anaplastic thyroid carcinoma, Chemotherapy, Docetaxel, Cisplatin

## Abstract

Anaplastic thyroid carcinoma has a dismal prognosis and lacks an established therapeutic strategy. We have recently conducted chemotherapy with docetaxel and cisplatin as part of multimodal treatment for eight patients with anaplastic thyroid carcinoma. Docetaxel (75 mg/m²) and cisplatin (75 mg/m²) were administered on day 1 every 4 weeks for six courses. This chemotherapy was used as induction therapy in one patient, as therapy for distant metastases in five patients and as postoperative adjuvant therapy in two patients. Three patients showed partial responses and three patients showed stable disease. After excluding the two patients receiving the treatment as adjuvant therapy the response rate was 50 %. Grade 3 or 4 leukocytopenia occurred in seven patients (88 %), but these adverse events were tolerable. Chemotherapy with docetaxel and cisplatin may thus be feasible for anaplastic thyroid carcinoma.

## Introduction

Anaplastic thyroid carcinoma (ATC) is rare, but it is one of the most aggressive and lethal types of cancer. The median overall survival is only 2–12 months, and the 1-year survival rate ranges from 9 to 46 % even with aggressive multimodal treatment [1–6].

While locally curative surgery and radiation prolong the survival in patients with stage IVA or B disease, the majority of these patients develop distant metastasis after local therapy. The survival of patients with stage IVC disease is even poorer. Systemic chemotherapy is, therefore, required to improve the patient survival [1].

Some investigators have recently reported that the use of taxanes, such as paclitaxel and docetaxel, resulted in higher response rates than those demonstrated by previous protocols [7–9]. The combination of docetaxel and cisplatin (DC therapy) was originally employed for patients with head-and-neck squamous cell cancer, and achieved good responses and tolerable adverse effects [10]. We have utilized DC therapy as a component of multimodal treatment for eight ATC patients at our institution since 2008, and herein present the outcomes of this treatment.

## Regimen of DC therapy

Docetaxel (75 mg/m²) and cisplatin (75 mg/m²) were administered intravenously on day 1 every 4 weeks for six courses. Premedication was performed in all patients using 8 mg of dexamethasone and 5-hydroxytryptamine receptor antagonists. We considered a reduction of the dose of both cisplatin and docetaxel to 80 % if we detected the creatinine clearance to be <60, or grade 1 renal dysfunction (assessed by creatinine), and a prolongation of myelosuppression or febrile neutropenia (FN), and also a low (one and more) performance status (PS). When grade 2 renal dysfunction occurred, then cisplatin was not utilized in the next course.

This regimen was approved by our institutional review board.

## Case reports

All patients were diagnosed with ATC based on pathological examinations, and all of the patients provided informed consent before receiving DC therapy. The clinical response was assessed by computed tomography (CT) according to the Response Evaluation Criteria in Solid Tumors version 1.1 after three or four courses of therapy. Adverse events were evaluated according to the Common Terminology Criteria for Adverse Events version 4.0. The time to progression was calculated from the initiation of DC therapy. The survival time in response to multimodal therapy, including DC therapy, was calculated from the time of initial therapy until the date of death. The characteristics of the eight cases are summarized in Table 1. All adverse events are summarized in Table 2.

**Table 1:** The clinical courses of eight patients who received chemotherapy with docetaxel and cisplatin as part of multimodal treatment

No.	Age	Sex	PS	Stage (TMN)	Surgery	Radiation	Objective of DC therapy	Response	Courses^a^	Survival (weeks)	TTP (weeks)
1	64	M	0	IVB (T4bN1bM0)	TT+ND	60 Gy	Postoperative adjuvant	NE	5	180	157
2	62	F	0	LN^(b)^	ND	60 Gy	Postoperative adjuvant	NE	6	280	^d^
3	58	F	0	IVC (T4bN1bM1)	LT+ND	–	Induction chemotherapy	PR	4	27	21
4	47	M	0	LN^b^	ND	60 Gy	Distant metastasis	SD	6	42	29
5	70	M	0	IVB (T4bN0M0)	LT+ND	CRT 40 Gy	Distant metastasis	PR	5	91	24
6	58	M	0	IVC (T4bN1bM1)	LT	–	Distant metastasis	PR	6^c^	227	^d^
7	70	M	1	IVC (T4bN1bM1)	TT+ND	–	Distant metastasis	SD	5	52	23
8	72	F	0	IVC (T4bN1bM1)	TT+ND	CRT 40 Gy	Distant metastasis	SD	6	174	^d^

*PS* performance status, *TT* total thyroidectomy, *LT* lobectomy, *ND* neck dissection, *CRT* chemoradiotherapy consisted of 40 Gy irradiation plus cisplatin, 5-fluorouracil and doxorubicin, *TTP* time to progression, *NE* not evaluable, *PR* partial response, *SD* stable disease

^a^Number of courses of chemotherapy completed

^b^Metastatic anaplastic cancer component in lymph nodes

^c^After six cycles of DC therapy, this patient was switched to single-agent docetaxel every 4 weeks for 30 cycles

^d^No progression

**Table 2:** Adverse events associated with combination chemotherapy with docetaxel and cisplatin

Adverse event	Grade 1 (number of patients)	Grade 2 (number of patients)	Grade 3 (number of patients)	Grade 4 (number of patients)	Ratio of G3+G4 (%)
Leukocytopenia	1	0	7	0	88
Neutropenia	0	1	4	3	88
Anemia	1	3	3	0	38
Thrombocytopenia	0	1	0	0	0
Febrile neutropenia	0	0	3	0	38
Diarrhea	0	0	2	0	25
Vomiting	0	0	1	0	13
Anorexia	3	3	1	0	13
Stomatitis	1	0	0	0	0
Renal dysfunction	3	2	0	0	0
Peripheral sensory neuropathy	2	0	0	0	0
Allergic reaction	0	1	0	0	0
Alopecia	1	0	0	0	0

### Case 1

A 64-year-old male presented with a 40-mm ATC in the right lobe of the thyroid. The tumor involved the tracheal wall and recurrent laryngeal nerve, and a 30-mm lymph node metastasis was identified at level IV in the right neck. We performed locally curative surgery, including shaving of the tracheal wall and recurrent nerve resection, and also administered 60 Gy of postoperative external beam radiation (EBRT). DC therapy was carried out as adjuvant therapy. An 80 % reduction in doses was performed after three courses, and the therapy was completely withdrawn after five courses because of FN. The patient survived for 3 years after the initiation of DC therapy without recurrence, then local recurrence developed and he died of the disease.

### Case 2

A 62-year-old female presented with an ATC component in a lymph node (level III; diameter, 30 mm) four years after lobectomy for papillary thyroid carcinoma (PTC). She underwent neck dissection and received 60 Gy of postoperative EBRT, then, DC therapy was initiated as adjuvant therapy. She completed six courses and remains alive without recurrence as of 5 years and 4 months after neck dissection, and 5 years after the initiation of DC therapy.

### Case 3

**Fig. 1:**
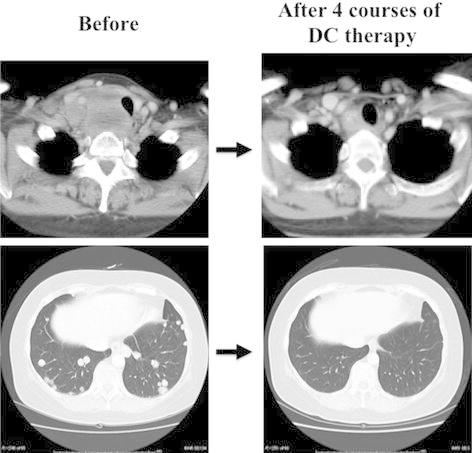
Computed tomography at the thyroid level and lung in a patient who received chemotherapy with docetaxel and cisplatin (DC therapy) as induction therapy (Case 3). The primary thyroid lesion showed a partial response, and the lung metastasis showed a complete response

A 58-year-old female presented with a 53-mm ATC in the right thyroid lobe that had invaded into the superior mediastinum and multiple lung metastases. We initiated DC therapy for this patient as induction chemotherapy. The patient showed a partial response (PR) for the thyroid lesion and a complete response (CR) for the lung lesions after four courses of therapy (Fig. 1). We then performed locally curative surgery, including resection of the right recurrent laryngeal nerve, and shaving of the trachea and esophagus, resection of the cicatrices around the right internal jugular vein and prevertebral muscles. However, the patient presented with convulsions and disturbance of consciousness 1 week after surgery, and brain metastases were identified on CT. We performed cranial irradiation and she recovered thereafter. However, she subsequently developed bone, liver, and skin metastases and both a recurrence of lung metastasis and local recurrence. She died of disseminated intravascular coagulation 6 months after the initiation of DC therapy, and 3 months after surgery.

### Case 4

A male patient had undergone total thyroidectomy for PTC when he was 5-year old. He developed multiple lung metastases when he was 22-year old, and underwent radioactive iodine therapies. The lung metastasis remained, but had not progressed after therapy. He presented with rapid enlargement of a right level III lymph node (diameter 51 mm) at age 43, and an ATC component was detected. We performed neck dissection and administered 60 Gy of postoperative EBRT. The lung metastasis showed rapid progression 3 months later, so DC therapy was initiated. The lung metastasis was defined as stable disease (SD) after three courses, and six courses were completed. A month after stopping DC therapy, the lung metastasis progressed rapidly. He died 7 months (30 weeks) after the initiation of DC therapy, which was 9 months (42 weeks) after surgery.

### Case 5

**Fig. 2:**
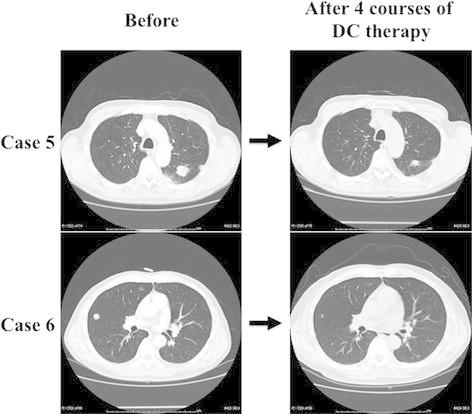
Computed tomography of the lungs in two cases with a partial response. Case 5 showed a 40 % reduction in the tumor volume from baseline. Case 6 showed a near-complete response

A 70-year-old male presented with a 40-mm ATC in the right thyroid lobe. Locally curative surgery was performed, including shaving of the trachea and esophagus, and resection of the right recurrent laryngeal nerve. We initiated postoperative concurrent chemoradiotherapy (CRT) that consisted of 40 Gy irradiation plus low-dose cisplatin (5 mg/m^2^) on days 1–5, 8–12, 15–19 and 22–26, 5-fluorouracil (200 mg/m^2^) on days 1–26, and doxorubicin (20 mg/m^2^) on days 1 and 15 [11]. Fourteen months postoperatively, we detected multiple lung nodules and a pathological examination showed anaplastic thyroid carcinoma component in those nodule, and, therefore, DC therapy was initiated. He underwent five courses of DC therapy, but cisplatin was not utilized in the fifth course because of grade 2 renal dysfunction. The patient showed a PR for the lung metastasis after the fourth course (Fig. 2), but progressive disease (PD) developed after the fifth course. He died of a progression of lung metastasis and survived for 1 year and 9 months (91 weeks) after surgery, which was 6 months (27 weeks) after the initiation of DC therapy.

### Case 6

A 58-year-old male presented with a 58-mm ATC in the left thyroid lobe and an 18-mm lymph node metastasis at level IV in the left neck. He also showed multiple lung metastases, but these were limited to 12 mm or smaller in diameter, so we performed locally curative surgery, including shaving of the tracheal wall and esophagus, and resection of the left recurrent nerve and internal jugular vein. The multiple lung metastases showed rapid progression with CT and high level accumulation with ^18^F-fluorodeoxy glucose positron emission tomography (FDG-PET) a month after surgery, so we assessed these metastases to be ATC and, therefore, administered DC therapy. He showed a PR for the lung metastases after four courses of DC therapy (Fig. 2), and six courses were completed, but cisplatin was not utilized in the sixth course because of grade 2 renal dysfunction. The patient has continued docetaxel monotherapy every 4 weeks and has remained alive for 4 years and 4 months after surgery (227 weeks), and 4 years and 3 months after the initiation of DC therapy.

### Case 7

A 70-year-old male presented with a 10-mm thyroid primary tumor in the left lobe, bilateral neck lymph node metastases up to 50 mm in diameter that involved the left common carotid artery and multiple lung metastases. We performed locally curative surgery, including the careful dissection of the carotid artery, and resection of the left recurrent nerve, internal jugular vein and left clavicle. The postoperative pathological examination revealed that both the primary thyroid tumor and lymph node metastasis were ATC. Bone metastases in the thoracic vertebrae were identified with FDG-PET 2 months after surgery, and DC therapy was initiated with an 80 % reduction of the doses because of PS1. The patient showed SD after three courses, but PD after five courses. He thereafter developed lung metastasis and died at 1 year (52 weeks) after surgery, which was 8 months (38 weeks) after the initiation of DC therapy.

### Case 8

A 72-year-old female presented with a 20-mm papillary thyroid carcinoma in the right thyroid lobe and bilateral neck lymph node metastases, and multiple lung metastases up to 10 mm in diameter were also detected. She underwent locally curative surgery, including partial resection of the tracheal wall and resection of the recurrent laryngeal nerve. The pathology showed an anaplastic cancer component only in a lymph node. We performed postoperative CRT comprising 40 Gy of irradiation plus low-dose cisplatin, 5-fluorouracil, and doxorubicin [11], but multiple lung metastases showed a rapid enlargement and high level accumulation on FDG-PET, and these metastases were identified as ATC at 5 months after surgery. We initiated DC therapy for the lung metastasis with an 80 % dose reduction because of grade 1 renal dysfunction due to the previous CRT. The patient showed SD after three courses and completed six total courses. The patient remains alive, and the lung metastases have been stable for 2 years and 10 months (148 weeks) after the initiation of DC therapy, which was 3 years and 4 months (174 weeks) after surgery.

## Discussion

In this report, DC therapy was used in three different ways, as: (1) adjuvant chemotherapy after local therapy (Cases 1 and 2); (2) induction chemotherapy (Case 3); and (3) chemotherapy for distant metastases after local therapy (Cases 4–8). Accurately assessing the efficacy of each type of therapy is difficult, but a PR was seen in three patients and SD was seen in three patients. After excluding the two patients who received DC therapy as adjuvant therapy, the response rate (PR) was 50 %, and the disease control rate (PR+SD) was 100 %. The two patients who received DC therapy as adjuvant chemotherapy were not evaluable (NE), but one survived as long as 180 weeks and the other is still alive without disease for more than 280 weeks. The clinical response and survival on DC therapy as components of multimodal treatment for these eight cases, which included stage IVB and C disease, appear to be as good as those in past studies using doxorubicin, cisplatin and taxanes [7–13]. Based on our findings, we considered it advantageous to add cisplatin to docetaxel monotherapy [9].

In case 3, the period from surgery until the development of new brain metastasis was very short. There is a possibility that blood–brain-barrier interferes with the effect of DC therapy in the brain. As a result, CT examinations are mandatory before starting DC therapy.

In case 5, the patient showed PR in the lesions of lung metastasis that developed after CRT using cisplatin, 5-fluorouracil and doxorubicin. This suggested the efficacy of DC therapy in CRT-resistant cases.

In case 8, the patient has survived a long time with lung metastasis, but without chemotherapy, after undergoing DC therapy. We determined these lung lesions to be metastasis of anaplastic thyroid carcinoma because of their rapid growth, but no pathological examinations were performed in this case. We, therefore, cannot definitively confirm that DC therapy eradicated the ATC components and there is possibility that these lung metastases were not ATC, but instead poorly differentiated papillary carcinoma.

One drawback associated with DC therapy is the high rate of leukocytopenia and neutropenia. In this report, grade 3 or 4 leukocytopenia and neutropenia occurred in seven patients (88 %). Furthermore, three patients developed FN, although, all recovered after the initiation of antibiotic therapy.

Four patients (50 %) completed six courses of therapy, but the remaining four patients dropped out after four or five courses. The reasons for the discontinuation were PD in three patients and adverse events in one patient. In two patients, cisplatin was discontinued after four or five courses of therapy due to grade 2 renal dysfunction; these patients were subsequently treated with docetaxel monotherapy. The doses of both cisplatin and docetaxel were 80 % of the intended dose in three patients because of renal dysfunction and an unfavorable performance status or adverse events. The completion rate of DC therapy thus appears to be insufficient.

Most patients with ATC experience rapid progression and some of these patients cannot tolerate aggressive treatment because of their poor general condition. Careful consideration of the balance between the individual biological malignancy of ATC and the intensity of treatment is thus important [14, 15]. It is important to emphasize that DC therapy is a high-intensity treatment that should be reserved for patients who are in good general condition and have a high PS.
